# Developmental trajectories of balance performance in preschoolers: age and gender differences for 3–6 years old Chinese children

**DOI:** 10.3389/fphys.2025.1615581

**Published:** 2025-07-23

**Authors:** Ruiyuan Li, Jiefeng Zhu, Ruiqin Li, Xiaoting Wang, Taishan Tian, Bingjun Wan

**Affiliations:** ^1^School of Physical Education, Shaanxi Normal University, Xi’an, China; ^2^Department of Physical Education, Xinzhou Normal University, Xinzhou, China; ^3^School of Physical Education, Shanxi Normal University, Taiyuan, China

**Keywords:** preschool children, postural control, balance performance, gender differences, age differences

## Abstract

**Objectives:**

Preschool age is a critical stage of postural balance development. Compromised stability may impede a child’s ability to acquire basic motor skills and, in turn, the capacity to participate in physical activities. However, little information exists on balance in preschool years, few studies have comprehensively evaluated all four balance types. in preschool children. Therefore, the purpose of the present study was to assess balance development in a multidimensional way in preschool children and to examine the effect of age and gender on preschool children’s balance performance.

**Methods:**

A total of 619 children (296 boys and 323 girls; aged 3–6 years) from China participated in the present study. Static steady-state, dynamic steady-state, proactive, and reactive balance performance were assessed using the one-leg stand test (OST), 10-meter walk test (10-MWT), functional reach test (FRT), and push and release test (PRT), respectively. Two-way analysis of variance was used to evaluate differences between age and gender groups.

**Results:**

The results revealed significant differences in gender for OST (η^2^_p_ = 0.037, *p* < 0.001), 10-MWT (η^2^_p_ = 0.012, *p* = 0.007), and FRT (η^2^_p_ = 0.016, *p* = 0.002). Age positively affected all balance tests, as the OST (η^2^_p_ = 0.336, *p* < 0.001), 10-MWT (η^2^_p_ = 0.448, *p* < 0.001), FRT (η^2^_p_ = 0.392, *p* < 0.001), and PRT (η^2^_p_ = 0.045, *p* < 0.001). Older preschool children performed better than their younger counterparts in all the tests. No significant interactions between age groups and gender were found.

**Conclusion:**

This study provided age- and gender-specific balance performance data in Chinese preschool children. All various balance types increased with age in this cohort. Static steady-state, dynamic steady-state, and proactive balance develop faster compared to reactive balance. Gender differences in balance already exist at the preschool age. These findings can assist health, physical education, and school professionals in assessing and improving balance in preschoolers using multiple indicators for different types of balance and designing age and gender-appropriate balance tasks.

## 1 Introduction

Balance is the ability to maintain postural control during both static and dynamic situations (Mickle et al., 2011). Sufficient balance is an essential basic prerequisite to competently undertake various activities of everyday daily life (e.g., standing, walking) (Shumway-Cook and Woollacott, 1985), and important for proficiency in movement skills. Given its fundamental role, early childhood is considered as a critical period of balance development (Shumway-Cook and Woollacott, 1985; Jiang et al., 2018). Balance depends on the integration of visual, vestibular, and proprioceptive inputs. These systems trigger muscular responses that produce postural adjustments (Goulème et al., 2018). Indeed, proprioceptive functions mature at 3–4 years (Steindl et al., 2006), motor control structures develop between 2–7 years (Cumberworth et al., 2007), and the vestibular system continues developing until age 10 (Cherng et al., 2001). At ages 3- to 6-years-old, children begin to appropriately use and integrate three different sources of sensory information (i.e., visual, vestibular, and proprioceptive) to maintain balance (Foudriat et al., 1993). At the age of 7 years, the neural circuits controlling posture mature (Ajrezo et al., 2013) By this age, the mechanisms of balance adjustment in children are similar to those in adults (Riach and Hayes, 1987; Schmid et al., 2005). Critically, early deficits in balance development may hinder the acquisition of fundamental movement skills and limit participation in physical activities later in life (King-Dowling et al., 2020).

Although balance development is crucial during the preschool period, few studies have investigated it comprehensively in healthy children. According to Shumway-Cook et al. (2017), balance includes four distinct types: static steady-state (i.e., maintaining a stable position while standing/sitting), dynamic steady-state (i.e., maintaining a stable posture while walking), proactive (i.e., the anticipation of predicted postural disturbances), and reactive (i.e., compensation for unpredictable postural disorders) balance. Many studies have been investigated balance performance in various populations according to this classification, such as athletes, youth (Schedler et al., 2020), adult (Granacher and Gollhofer, 2011), as well as the elderly (Muehlbauer et al., 2012). Although several studies have used this classification to assess balance in various populations, no studies to date has comprehensively examined all four balance types in preschool children. Additionally, although balance developing altered with age, postural balance performance may not be uniform throughout the whole preschool period (Verbecque et al., 2016). There is a need to further explore the age-specific characteristics of balance in preschoolers.

Furthermore, regarding gender effect, some controversial results are mentioned in the literature. Some papers have noted no differences between boys and girls in static or dynamic balance (Kakebeeke et al., 2012; Latorre-Roman et al., 2021). For instance, Kasuga et al. (2012) found that the difference in walking time between boys and girls was not significant and there was no gender difference. However, other studies found that girls have better static or dynamic balance performances than boys (Deoreo and Wade, 1971; Lee and Lin, 2007; Shams et al., 2020). Specifically, Smith et al. (2012) found that when it comes to sensory information use, girls and boys act differently in term of strategies. Mnejja et al. (2022) suggested that girls aged 4–5 years were better able to integrate vestibular sensory information to maintain postural balance as compared to boys. Gender differences in balance performance of preschool children remain unclear. Moreover, no study has examined gender differences specifically in dynamic steady-state and reactive balance among preschool children. It seems need to further explore the gender effect on preschoolers’ balance abilities.

Therefore, this study aimed to comprehensively assess balance developmental trajectory in healthy preschoolers and determine its moderation by age and gender. It was hypothesized that balance ability improved with age. Based on evidence that balance is task-specific (Kiss et al., 2018), we further hypothesized age-specific differences across the four balance types. A secondary purpose of this study was to examine gender effects on balance performance. We hypothesized that girls would outperform boys in balance performance, as they employ more mature balance strategies earlier (Kolic et al., 2020).

## 2 Materials and methods

### 2.1 Participants

This is a cross-sectional study. The sample size (N = 225) was determined *a priori* using G*Power (version 3.1.9.7; Franz Faul, University of Kiel, Germany) to estimate the minimum required sample size for a two-factor ANOVA design, with α = 0.05; power = 0.8; effect size (f) = 0.25. Three public kindergartens in Beijing, China, were selected via convenience sampling. Within these institutions, participants were stratified by gender and age group, and then randomly selected from each stratum using a computer-generated randomization process (296 boys and 323 girls; aged 3–6 years) (Urbaniak, 2021; GCU, 2021). Participant characteristics are detailed in Table 1. Inclusion criteria were: 1) typical physical development with no major illnesses or physical disabilities; 2) no recent trauma affecting physical activity participation 3) no acute illness (e.g., cold or fever); or ongoing medical treatment affecting balance during testing; 4) capacity to follow simple instructions. Participation in the balance tests was voluntary. The classification of age groups was based on the principles and descriptions of the CNPFM-Pre (China, 2003). The 3-year-old group included children aged 3.0–3.49 years; the 3.5-year-old group included children aged 3.5–3.99 years; the 4-year-old group included children aged 4.0–4.49 years; the 4.5-year-old group included children aged 4.5–4.99 years; the 5-year-old group included children aged 5.0–5.49 years; and the 5.5-year-old group included children aged 5.5–5.99 years. Before conducting the test, a detailed explanation was provided to the parents regarding the aims and risks associated with the investigation, with assistance from the kindergarten management. Parents were informed of their right to withdraw their child at any time without consequences, and written informed consent was procured from the parents. The study was approved by the Ethics Committee of Beijing Sport University Institutional Research Commission (Approval number: 2022155H), and the study procedures were performed in accordance with the Declaration of Helsinki.

**TABLE 1:** Descriptive characteristics of the participants.

Age (years)	All				Boys				Girls			
	n	Height (cm)	Weight (kg)	BMI (kg/m^2^)	n	Height (cm)	Weight (kg)	BMI (kg/m^2^)	n	Height (cm)	Weight (kg)	BMI (kg/m^2^)
3-year-old group	82	100.10 ± 3.79	15.44 ± 1.70	15.43 ± 1.25	41	99.85 ± 3.97	15.59 ± 1.85	15.61 ± 1.22	41	100.35 ± 3.63	15.30 ± 1.55	15.24 ± 1.26
3.5-year-old group	102	103.45 ± 3.62	16.32 ± 1.67	15.25 ± 1.17	45	103.42 ± 3.47	16.55 ± 1.61	15.47 ± 1.12	57	103.48 ± 3.76	16.14 ± 1.71	15.07 ± 1.19
4-year-old group	99	108.01 ± 4.14	17.94 ± 2.07	15.37 ± 1.16	53	109.38 ± 4.42	18.69 ± 2.24	15.60 ± 1.36	46	106.43 ± 3.15	17.07 ± 1.43	15.11 ± 0.80
4.5-year-old group	130	110.33 ± 4.52	18.78 ± 2.22	15.40 ± 1.30	63	110.28 ± 4.84	18.70 ± 2.26	15.30 ± 1.20	67	110.38 ± 4.23	18.86 ± 2.20	15.49 ± 1.40
5-year-old group	105	114.05 ± 4.48	19.88 ± 2.39	15.30 ± 1.42	47	115.02 ± 4.62	20.43 ± 2.08	15.45 ± 1.43	58	113.26 ± 4.24	19.44 ± 2.55	15.17 ± 1.42
5.5-year-old group	101	117.26 ± 4.72	21.25 ± 3.29	15.39 ± 1.50	47	117.71 ± 4.87	21.60 ± 3.67	15.55 ± 1.67	54	116.87 ± 4.59	20.94 ± 2.92	15.24 ± 1.34
Total	619	108.87 ± 4.21	18.27 ± 2.22	15.35 ± 1.31	296	109.28 ± 4.37	18.59 ± 2.29	15.49 ± 1.34	323	108.46 ± 3.93	17.96 ± 2.06	15.23 ± 1.27

### 2.2 Procedures

Before conducting the balance tests, body weight (kg) and height (cm) of the participants were measured without shoes and coats using a balance scale (V-BODY HBF-371, Omron, Japan) and a stadiometer (Ningbo Finer Medical Instruments Co., Limited, Zhejiang, China), respectively. Measurements were taken between 8:00 and 10:00 a.m. after an overnight fast, with no vigorous physical activity in the preceding 2 h. Thereafter, their body mass index was calculated (weight/height squared [kg/m^2^]). All the tests were conducted in a large kindergarten classroom during morning, and the children were tested in small groups (4 children per group). The order of the four tests was randomized, and they were conducted on the same day. The complete assessment protocol required approximately 45 min per child, including warm-up, practice trials, and formal testing. The children performed a moderate warm-up exercise that primarily involved jogging and aerobic exercises before participating in the tests (Alvarez et al., 2008). Thereafter, the examiner carefully explained the test procedures to the participants, specified the test requirements, and demonstrated how to perform the tests, ensuring they understood the details of the test. After this, the children were familiarized with each test and were allowed two practice attempts on each test. If a child did not understand the task to perform it appropriately, the testing procedures were explained again and the demonstration was repeated. Two or three formal balance tests were conducted for each test item. A test taker was responsible for recording the children’s test scores on the test list immediately after the children completed the test.

### 2.3 Balance test

#### 2.3.1 Static steady-state balance

##### 2.3.1.1 One-leg stand test

Static steady-state balance was measured using the one-leg stand test (OST). The timed one-leg standing measurement is used as an static balance measure and has shown good test-retest reliability in typically developing children (Atwater et al., 1990). Participants were asked to stand barefoot on their dominant leg as long as possible with standardized posture. The stopwatch is started as soon as one foot is lifted and stopped when the child loses balance or the child’s raised leg touches the floor. Trials were repeated if procedural errors occurred. After two practice trials, two formal tests were administered, and the duration for the test was recorded (in seconds, accurate to 0.01s). The best attempt was evaluated/or recorded for further evaluation. Dominance assessment details are published elsewhere (Li et al., 2024).

#### 2.3.2 Dynamic steady-state balance

##### 2.3.2.1 10-M walk test

Dynamic steady-state balance represented by gait speed was tested using the 10-meter walk test (10-MWT) (De Baptista et al., 2020; Rygelová et al., 2023). Gait speed, a critical gait parameter, has been extensively studied in preschool children. Walking tests based on walking speed have been shown to effectively reflect balance control in this age group (Guffey et al., 2016; Verbecque et al., 2017). A 12-m straight walkway was established. The walkway included a 1-m acceleration zone before the timed 10-meter section and a 1-m deceleration zone after it, with clearly marked lines at 0 m (start target line), 1 m (timing start line), and 11 m (timing finish line). After the child wears their footwear and stands naturally at the start target line (0 m line), when they hear the “Go” command, participants to “walk as fast as possible without running” (Schedler et al., 2021). Timing commenced when the lead foot crossed the 1 m line and ceased when any foot crossed the 11 m line. The initial 0–1 m section served as the acceleration zone and the final 11–12 m section as the deceleration zone; only the 1–11 m section (10 m) was timed for steady-state gait speed calculation. The time was accurate at 0.01 s. Gait speed (m/s) was then calculated and used for analysis. Two experimental trials were recorded, and the better one was used for further analyses.

#### 2.3.3 Proactive balance

##### 2.3.3.1 Functional reach test

Proactive balance was measured using the functional reach test (FRT). The test was assessed in typically developing children and was found to have high reliability (Norris et al., 2008). A baseline tape was placed perpendicular to the wall on the floor. Participants stood with toes positioned behind the tape, feet shoulder-width apart (medial malleoli 15–20 cm apart) and flat on the floor. The acromion process of the left shoulder was maintained at 15 cm from the wall, with the torso perpendicular to the wall and 0° shoulder rotation. A 1-m graduated straightedge was secured to the wall at the height of the acromion process (Bañas and Gorgon, 2014). The FRT was demonstrated and described as follows: “Clench your fist raise your arms to shoulder height (90° flexion). Reach forward as far as you can, but don't fall or step forward.” To measure the FR distance, the initial measurement was taken with the child’s arm raised horizontally (approximately 90° of shoulder flexion), using the position of the third metacarpal along the metric ruler. A second measure was taken after reaching, again using the location of the third metacarpal along the metric ruler. This procedure was performed three times, and the average was taken as the result.

#### 2.3.4 Reactive balance

##### 2.3.4.1 Push and release test

Reactive balance performance was measured using the push and release test (PRT), a field-based method for assessing reactive balance (Schedler et al., 2020). To ensure the validity and consistency of the test, the PRT is performed by a medical professional who completes the test on all children. Repeated tests were conducted prior to the formal test, and it showed acceptable test-repeat reliability (Table 2). The child stood barefoot with their back to the tester, who placed their hand on the child’s scapula. The child leaned backward against the tester’s palm, continuing until their shoulders and hips moved behind their heels. At that point, the tester quickly removed their hand to observe the child’s ability to recover balance. Performance was scored based on recovery steps and quality. The judgment criteria were as follows: 0 points = 1 step, 1 point = independent recovery after 2 - 3 small steps, 2 points = self-sustaining recovery after ≥4 steps, 3 points = need for multiple steps with assistance to recover, and 4 points = falling or unable to stand without assistance. To ensure scoring objectivity, all tests were simultaneously observed and independently scored by two trained raters using standardized criteria, with discrepancies resolved through immediate discussion. The test was performed twice, and the best of the two attempts was recorded.

**TABLE 2:** Intraclass correlation coefficients (ICC) for all balance measures.

Variable (measure)	ICC	ICC 95% CI
OST (static steady-state balance)	0.96	(0.935, 0.976)
10MWT (dynamic steady-state balance)	0.85	(0.767, 0.910)
FRT(proactive balance)	0.98	(0.979, 0.992)
PRT(reactive balance)	0.86	(0.779, 0.915)

*Note*. OST, one-leg stand test, 10MWT, 10-meter walk test, FRT, functional reach test; PRT, push and release; CI, confidence interval.

#### 2.3.5 Intraclass correlation coefficients

Intraclass correlation coefficients (ICC) were calculated for all balance measures. The results indicated robust test-retest reliability (Table 2).

### 2.4 Statistical analysis

Experimental data were processed using IBM SPSS statistical software package (version 26.0, Chicago, IL, United States). All data were presented as “mean ± standard deviation” (M±SD) values. Kolmogorov–Smirnov (with Lilliefors correction) and Levene’s tests were used to assess normality and homogeneity of variance, respectively. Firstly, a two-way analysis of variance (ANOVA) was applied to determine any significant (*p* ≤ 0.05) main effects of age (3, 3.5, 4, 4.5, 5, or 5.5 years) or gender (boy or girl) or age × gender interactions on the OST, 10-MWT, FRT and PRT results. When a significant interaction was observed, Tukey’s Honestly Significant Difference *post hoc* correction was performed to identify the specific interaction Secondly, one-way ANOVA were used to evaluate differences in the results among the age groups (3, 3.5, 4, 4.5, 5, and 5.5 years), followed by *post hoc* pairwise comparisons using the Games-Howell approach. *Partial η* ^2^ was used to determine the effect sizes (ES) when significance was observed, with its strength being interpreted as follows: <0.06, small; <0.14, moderate; and ≥0.14, large (Cohen, 1988). The level of significance was set at *p* < 0.05 for all tests.

The relative test reliability was assessed using the intraclass correlation coefficient of the one-way random-effects model with a single measure (i.e., ICC). Statistical significance was set at *p* < 0.05 for all tests.

## 3 Results

**FIGURE 1:**
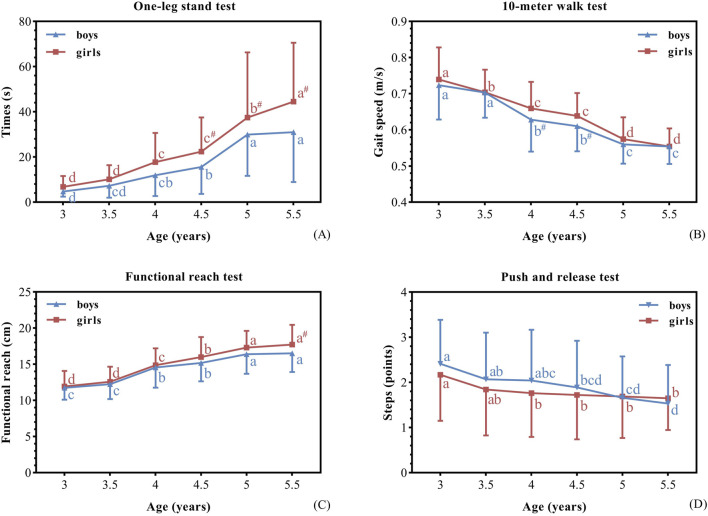
Balance performances of boys and girls. Balance performance (mean ± standard deviation) of boys and girls (using two-way ANOVA). The (#) indicates a significant difference between genders at p < 0.05; (a, b, c, d) is a letter-marking method used to compare differences between age groups, with identical letters indicating no significant differences. **(A)** one-leg stand test, **(B)** 10-meter walk test, **(C)** functional reach test, **(D)** push and release test. In the 10MWT, and PRT tests, smaller value indicated a better performance.

Table 3 and Figure 1 show the performance of participants on the different types of balance tests according to age and gender. Older preschool children performed better than the younger ones in all balance tests. Boys performed better than girls in dynamic steady-state balance (10-MWT). Girls performed better than boys in static steady-state balance (OST), and proactive balance (FRT).

**TABLE 3:** Results of balance tests according to gender and age.

Balacne tests	All (n = 619)	Boys (n = 296)	Girls (n = 323)	Sex		3-year-old group (95% CI)	3.5-year-old group (95% CI)	4-year-old group (95% CI)	4.5-year-old group (95% CI)	5-year-old group (95% CI)	5.5-year-old group (95% CI)	Age		Age ∗sex	
				P	η^2^_p_							P	η^2^_p_	P	η^2^_p_
OST (s)	20.56 ± 20.38	16.85 ± 16.59	23.96 ± 22.82*	<0.001	0.037	5.75 ± 3.86^d^	8.86 ± 5.97^cd^	14.57 ± 11.42^bc^	9.05 ± 14.06^b^	34.06 ± 24.86^a^	38.19 ± 25.12^a^	<0.001	0.340	0.179	0.012
						(4.90–6.60)	(7.68–10.03)	(12.30–16.85)	(16.61–21.49)	(29.25–38.87)	(33.23–43.15)				
10MWT (m/s)	6.34 ± 0.93	6.26 ± 0.95	6.40 ± 0.91*	0.007	0.012	0.73 ± 0.09^a^	0.71 ± 0.07^a^	0.64 ± 0.08^b^	0.63 ± 0.07^b^	0.57 ± 0.06^c^	0.55 ± 0.05^c^	<0.001	0.452	0.453	0.008
						(0.71–0.75)	(0.69–0.72)	(0.63–0.66)	(0.61–0.64)	(0.56–0.58)	(0.54–0.56)				
FRT (cm)	14.90 ± 3.14	14.55 ± 3.01	15.23 ± 3.23*	0.002	0.016	11.84 ± 1.89^c^	12.43 ± 2.07^c^	14.69 ± 2.59^b^	15.59 ± 2.69^b^	16.89 ± 2.53^a^	17.16 ± 2.71^a^	<0.001	0.392	0.645	0.006
						(11.42–12.25)	(12.02–12.84)	(14.18–15.21)	(15.13–16.06)	(16.40–17.38)	(16.62–17.69)				
PRT (pt)	1.85 ± 0.99	1.92 ± 1.03	1.79 ± 0.95	0.103	0.004	2.29 ± 1.00^a^	1.95 ± 1.02^ab^	1.90 ± 1.06^ab^	1.80 ± 1.01^b^	1.68 ± 0.92^b^	1.59 ± 0.78^b^	<0.001	0.046	0.626	0.006
						(2.08–2.50)	(1.77–2.14)	(1.71–2.10)	(1.64–1.97)	(1.49–1.86)	(1.40–1.78)				

*girls vs. boys, *p* < 0.05; a, b, c, and d is a letter-marking method used to compare differences between age groups, with identical letters indicating no significant differences; p-values indicate differences between settings using ANOVA, analyses. The data are shown as a mean ± SD. CI, confidence interval.

Firstly, the two-way ANOVA models revealed no significant interaction between age and gender on the OST (F = 1.527, *p* = 0.179), 10-MWT (F = 0.943, *p* = 0.453), FRT (F = 0.672, *p* = 0.645) and PRT (F = 0.697, *p* = 0.626). Further, it was found a significant main effect of age on different types of balance performance in terms of OST (F = 62.433, *p* < 0.001), 10-MWT (F = 100.31, *p* < 0.001), FRT (F = 78.361, *p* < 0.001), and PRT (F = 5.903, *p* < 0.001), Table 3.

Secondly, one-way ANOVA models showed that in terms of the OST, the 3.5-, 4-, and 5-year-old groups differed significantly from the 3-, 3.5-, and 4.5-year-old groups, respectively (*p* < 0.001). With regard to 10-MWT, significant differences were observed between groups of 3.5- and 4-year-olds, and 4.5- and 5-year-olds (*p <* 0.001). Regarding the FRT, the 4- and 5-year-old groups differed significantly from the 3.5- and 4.5-year-old groups, respectively (*p <* 0.001). Furthermore, a significant difference in the PRT scores was observed for 3.5-year-olds (*p =* 0.017) (see Table 3).

Further comparison of the differences in balance performance of boys and girls of each age group on the balance tests was performed. Boys performed significantly better than girls in the 10-MWT (4years [*p* = 0.026] and 4.5 years [*p* = 0.021]; see Figure 1). Girls performed significantly better than boys in the OST (4.5 years [*p* = 0.018], 5 years [*p* = 0.019], and 5.5 years [*p* < 0.001]) and FRT (5.5 years [*p* = 0.014]) tests (see Figure 1).

## 4 Discussion

This study provides age and gender developmental characteristics of different balance types (static steady-state, dynamic steady-state, proactive, and reactive balance) in preschool children. According to the hypotheses, the results showed that balance performance improved with age, as older preschoolers outperformed younger preschoolers in all balance tests (e.g., longer single-leg stance duration in 5- vs. 3-year-olds; shorter 10-meter walk time in older groups). Additionally, girls performed better than boys in static steady-state and proactive balance, whereas boys performed better than girls in dynamic steady-state balance. These findings can assist health, physical education, and school professionals in assessing and improving balance in preschoolers using multiple indicators that reflect different types of balance. The findings can also help stakeholders design appropriate balance tasks for boys and girls of different ages.

Consistent with our first hypothesis, we observed that older preschool children outperformed their younger counterparts in all balance test items. Specifically, significant differences were observed in static steady-state, dynamic steady-state, and proactive balance among 3-, 4-, and 5-year-olds, while 3.5-year-olds outperformed 3-year-olds and 5.5-year-olds outperformed 3.5-year-olds in reactive balance (Table 3). Additionally, the absence of significant differences between closely spaced age groups (e.g., 4- vs. 4.5-year-olds) may reflect slower balance maturation during transitional periods, aligning with reported nonlinear progression in motor development (Chen et al., 2010; Bisi et al., 2018). This suggests that balance ability increases with age in preschool children aged 3–6 years. This finding aligns with previous studies indicating that balance control improves with maturation (Cumberworth et al., 2007; Shams et al., 2020; García-Liñeira et al., 2021). Mnejja et al. (2022) found significantly better postural balance in 5-year-old *versus* 4-year-old Tunisian children during static standing trials. Similarly, Latorre-Román et al. (2021) reported progressive age-related improvements in Spanish preschoolers using the Balance Beam Test. Preschool children are in a period of continuous motor, physiological and body structure developmental changes (Berk, 2013). As their bodies develop with age increases, their nervous systems, sensory system, motor control systems, and motor patterns develop significantly (Tanaka et al., 2012), which may enhance their body posture stability and balance (Assaiante et al., 2005). These results are consistent with maturation of the central nervous system involved in the integration and use of sensory strategies in postural balance (Venetsanou and Kambas, 2011). Since the ability to process balance-related sensory signals develops between ages 3–6 years, younger children are less able to filter out distracting visual and body-position cues (Forssberg and Nashner, 1982), and their ability to use sensory compensatory strategy is lower as compared to older children (Cumberworth et al., 2007). As children grow in age, they become more capable of processing sensory manipulations, compensating for lost or interfered sensory inputs with precise sensory strategies. Further, younger children can be considered as “early in practice” with balance tasks, may face greater challenges compared to older children, who have more experience with such tasks due to older age and accumulated task-specific practice. Daily balance-challenging activities (e.g., hopping games for 3–4 year-olds to improve proactive balance; beam walking for 5–6 year-olds to enhance dynamic steady-state control) contribute to this developmental progression. This might explain the increase in balance with age. These improvements are fundamental for mastering basic motor skills like hopping and kicking, and for preventing falls during daily activities.

Additionally, in regard to age differences, the present study found that age-related growth patterns differed among the various types of balance in 3-6-year-olds. While all types of balance improved with age, the η^2^_p_ between age groups (Table 3) indicate that static steady-state, dynamic steady-state, and proactive balance develop more rapidly compared to reactive balance. This differential development likely occurs because reactive balance may involve later-maturing neuromotor processes in childhood. Moreover, the age characteristics were not identical even between static steady-state, dynamic steady-state, and proactive balance. Based on the view that balance is task-specific (Muehlbauer et al., 2013), it is reasonable to discovered distinct age-related growth characteristics among the various types of balance. The different ageing characteristics may be attributable to the different neurophysiological mechanisms required to engage in the specific postural control tasks (Granacher and Gollhofer, 2012; Verbecque et al., 2021). For example, Lau et al. found that connections involving the sensorimotor cortex was significantly greater while standing compared to walking (Lau et al., 2014), suggesting that standing requires more active cortical control to maintain balance and posture (Tokuno et al., 2009). In contrast, walking relies more on spinal neural networks for locomotor control (Lau et al., 2014). Moreover, proactive (i.e., FRT) and reactive (i.e., PRT) balance involve different mechanisms. Proactive balance relies substantially on feedforward control, including anticipating postural disturbances while leaning forward to the maximum extent and initiating sufficient muscle responses to prevent loss of balance. In contrast, reactive balance relies primarily on feedback control and is characterized by the initiation of sufficient muscle responses after loss of balance to compensate for unpredictable postural disturbances and avoid falls. Functionally, this mirrors bracing before sliding down a slide (proactive) *versus* recovering after tripping on a toy (reactive). In this context, Wälchli et al. (2017) and Fujio et al. (2018) showed that the central nervous system exhibits different predictive postural control strategies for expected *versus* unexpected postural perturbations. Specifically, the excitability of corticospinal pathway is muscular for unexpected postural perturbations and is modulated based on the current posture and anticipated future states (Fujio et al., 2018). Additionally, differences in balance task difficulty and complexity might also contribute to the observed distinct age-specific growth characteristics (Kiss et al., 2018) Dynamic balance tests seem to be more challenging than the static balance tests (Gonçalves et al., 2022). For example, in a static steady-state balance task (i.e., OST), only the center of gravity shifted while the body support points (i.e., foot) were stable, but in a dynamic steady-state balance tasks (i.e., walking) involve both the center of gravity and support point movement (Liu et al., 2024). This complexity could help explain the differences between types of balance abilities with age. The results indicating the need for complimentary testing and individual measurement of each type when assessing balance performance in preschool-aged children.

In accordance with our second hypothesis, we found girls showed better balance performances in most tests compared with that shown by boys (i.e., static steady-state, proactive, and reactive balance), especially older girls. However, for the PRT task, this difference was not statistically significant (Figure 1). In contrast, boys performed better in the tasks involving dynamic steady-state balance. Our study are in accordance with previous studies that observed better balance performance overall in girls (Shams et al., 2020; Heidt et al., 2021; Li et al., 2022). For example, Heidt et al. (2021) assessed children’s balance using a single 3D motion tracking camera and found gender differences with girls having better postural stability. Mnejja et al. (2022) also reported similar results that Tunisian girls had better postural balance than boys. These differences may be attributed to several possible factors, including earlier maturation of the central neural structures (Plandowska et al., 2019), the capable of visual and vestibular inputs (Goulème et al., 2018), the ability of sensory information integration (Peterson et al., 2006), and the use of more sophisticated postural control strategies (De Bellis et al., 2001). In this context, research on brain maturation has shown that the structure and development of young children’s brain differs between sexes (Lenroot and Giedd, 2006). The cerebral volume and gray matter in the frontal and parietal lobes peak earlier in girls than in boys, and central neural structures mature earlier (De Bellis et al., 2001). Mnejja et al. (2022) found that girls are more capable to cope with visual information inputs absence than boys. Other studie remarkable gender difference is that girls have greater postural control under motor conditions in which the vestibular system imputs information (Smith et al., 2012). Likewise, Peterson et al. (2006) also showed that girls are better at using vestibular sensory information and are more capable of integrating their senses. Once again, this result could be due to that girls can more efficient use of sensory strategies to compensate for challenged inputs while maintaining postural control compared to boys (Goulème et al., 2018). In the same way, Kolic and colleagues noted that girls employing more mature balance strategies at an earlier age, suggesting that girls perform better than boys in terms of balance (Kolic et al., 2020). Additionally, our study found that boys performed better than girls in 10-MWT (Figure 1) Findings are consistent with reports that boys outperform girls in complex dynamic balance tasks (Demura, 1995), such as walking on a balance beam (Venetsanou and Kambas, 2011). Thus, boys may have an advantage over girls in dynamic balance because they can leverage strength advantages to optimize dynamic balance control (Schedler et al., 2019). Whereas the absence of significant gender differences in PRT (Figure 1) likely reflects preschool-specific maturation patterns; future studies should investigate gender effects in 6-8-year-olds to define age-specific progression of reactive balance control, complemented by instrumented motion analysis for multidimensional profiling of neuromuscular dynamics. Moreover, Jiang et al. (2018) reported that boys preferred vigorous physical activities, whereas girls participated more in activities such as dance and gymnastics; the authors proposed that such activities could enhance balance development. It is important to note that, although previous studies have considered gender differences in balance in preschoolers, the data provided were extracted from a small sample size. Further studies are needed in order to corroborate or contrast these findings. Furthermore, other factors that could explain these differences are attention allocation, motivation, or different methods of assessing balance tests in the study. This highlighting the need for multiple indicator measures to assess balance in preschool children.

An important strength of this study is its multidimensional assessment of four distinct types of balance ability (static steady-state, dynamic steady-state, proactive, and reactive balance) in a large sample of preschool children, utilizing ecologically valid field-based methods. This comprehensive approach not only provides data on age and gender differences in balance development but also supplements the current evidence base with valuable empirical reference values for future research. To our knowledge, the work is the first to report on the age and gender-related characteristics of different types of balance performance in such a large sample of preschool children. Unlike previous studies, our findings indicate that while balance develops gradually with age, each type of balance in 3-6-year-olds showed distinct, age-specific growth characteristics. In addition, girls demonstrated better balance (i.e., static steady-state, proactive, and reactive balance) overall in the tests compared to boys, whereas boys outperformed girls in dynamic steady-state balance. The use of these field-based methods to assess balance in preschoolers is another key strength, as they can be easily implemented in authentic venues, such as schools, without requiring specific instruments. This approach provides significant value for large-scale testing of preschool children.

However, some limitations are associated with this study. First, this study used a cross-sectional design to measure balance performance. While providing age-group comparisons, it cannot track individual developmental changes over time. Future longitudinal studies are needed to observe the changes within the same children over time and to validate potential causal relationships underlying the age and gender differences identified in this study. Secondly, the participants were all children from one geographical region. Environmental or socio-cultural factors in other regions might differently influence balance development. Future studies involving children from diverse regions are needed to validate and broaden the applicability of these observations. Thirdly, while the focus was on age and gender, other factors such as physical activity levels, nutrition, socioeconomic status, or habitual movement patterns may also influence balance performance. Future studies should develop dedicated protocols to assess these variables, elucidating their specific mechanistic roles.

## 5 Conclusion

This study delineates the developmental status of different balance types in Chinese preschool children aged 3–6 years, with performance compared across gender and age. The main findings of this study are that balance performance develops gradually with age during the preschool period, with each balance type exhibiting distinct age-specific growth characteristics. Similarly, studies have reported gender differences in balance among preschoolers. Specifically, older girls demonstrated better performance in static steady-state (e.g., standing stability) and proactive balance (e.g., anticipatory adjustments), while boys performed better than girls in dynamic steady-state balance (e.g., movement stabilization). These findings can provide guidance to health, physical education, and school professionals to design balanced assessments and training programs. To implement this effectively, a multiple-indicator approach is recommended to evaluate preschool children’s balance abilities and incorporate exercises targeting all types of balance. Furthermore, age- and gender-appropriate balance tasks should be designed in accordance with the developmental stages to optimize training outcomes.

## Data Availability

The original contributions presented in the study are included in the article/supplementary material, further inquiries can be directed to the corresponding authors.
